# Diabetes mellitus and prognosis in women with breast cancer: A systematic review and meta-analysis

**DOI:** 10.1097/MD.0000000000005602

**Published:** 2016-12-09

**Authors:** Xiao-Bo Zhao, Guo-Sheng Ren

**Affiliations:** a Chongqing Key Laboratory of Molecular Oncology and Epigenetics, The First Affiliated Hospital of Chongqing Medical University, Chongqing; b Department of Breast Surgery, Affiliated Hospital of North Sichuan Medical College, Nanchong; c Department of Endocrine and Breast Surgery, The First Affiliated Hospital of Chongqing Medical University, Chongqing, China.

**Keywords:** breast cancer, diabetes mellitus, meta-analysis, prognosis

## Abstract

**Background:**

Diabetes mellitus is associated with an increased risk of breast cancer, but studies of the effects of diabetes on the prognosis of women with breast cancer have yielded inconsistent findings. The present meta-analysis aimed to investigate the impact of preexisting diabetes on the prognosis in terms of overall survival (OS), disease-free survival (DFS), and relapse-free period (RFP) in women with breast cancer.

**Methods:**

We searched the Embase and PubMed databases until June 2016 for cohort or case–control studies assessing the impact of diabetes on the prognosis of women with breast cancer. The pooled multivariate adjusted hazard ratio (HR) and their 95% confidence intervals (CIs) for OS, DFS, and RFP were used to analyze the impact of diabetes on the prognosis of breast cancer patients.

**Results:**

Seventeen studies involving 48,315 women with breast cancer met our predefined inclusion criteria. Meta-analysis showed that the pooled adjusted HR was 1.51 (95% CI 1.34–1.70) for OS and 1.28 (95% CI 1.09–1.50) for DFS in breast cancer patients with diabetes compared to those without diabetes. However, RFP did not differ significantly between patients with and without diabetes (HR 1.42; 95% CI 0.90–2.23).

**Conclusions:**

The present meta-analysis suggests that preexisting diabetes is independently associated with poor OS and DFS in female breast cancer patients. However, the impact of diabetes on RFP should be further verified. More prospective studies are warranted to investigate whether appropriate glycemic control with modification of antihyperglycemic agents can improve the prognosis of female breast cancer patients with diabetes.

## 1 Introduction

Breast cancer remains one of the most common neoplasms and a major cause of cancer-related death among women.^[1]^ Diabetes mellitus is an increasing global public health concern.^[2]^ A total of 382 million people had diabetes in 2013 and this number is expected to rise to 592 million by 2035.^[3]^ Diabetes and breast cancer are quite prevalent chronic diseases among women. Approximately 16% of breast cancer patients suffered from diabetes.^[4]^ The coexistence of diabetes in addition to breast cancer could alter treatment regimens and chemotherapy toxicity,^[5]^ subsequently negatively affecting the prognosis.^[6]^

Diabetes mellitus has been identified as an independent risk factor for breast cancer.^[7]^ The effect of diabetes on the prognosis of breast cancer patients has been extensively investigated in recent years. A well-designed meta-analysis showed that women with diabetes had a 23% greater risk of subsequent breast cancer than those without diabetes.^[8]^ Another recently published meta-analysis found that preexisting diabetes is associated with a 37% and 17% greater risk of all-cause mortality and breast cancer mortality in female breast cancer patients, respectively.^[9]^ However, these 2 meta-analyses^[8,9]^ did not examine the impact of diabetes on breast cancer prognosis in terms of disease-free survival (DFS) and relapse-free period (RFP). DFS and RFP are potential candidates as surrogates for overall survival (OS) in the clinical setting. The treatment effect on the OS can be predicted according to the DFS or RFP, thus reducing the trial duration. Individual studies on the association between diabetes and breast cancer prognosis have yielded inconsistent results.^[10]^ These conflicting findings may be confounded by diabetes treatments, particularly the use of metformin.^[11]^

Here, we conducted a meta-analysis of the available observational studies to investigate the impact of preexisting diabetes on the OS, DFS, and RFP in women with breast cancer.

## 2 Methods

### 2.1 Search strategies

The present meta-analysis was performed according to the checklist of the Meta-Analysis of Observational Studies in Epidemiology.^[12]^ The present meta-analysis of already published data did not require ethics committee approval or patient consent. Two reviewers (X-BZ and G-SR) independently conducted a comprehensive literature search of the PubMed and Embase databases from their inception to June 2016, for articles that evaluated the impact of diabetes on the clinical prognosis of breast cancer patients. The search terms used were (diabetes OR diabetic OR hyperglycemia) AND (breast cancer OR breast carcinoma OR breast neoplasm) AND (prognosis OR survival OR relapse OR recurrence OR mortality OR death). In addition, the references cited in the relevant articles were manually reviewed to identify any additional articles. The literature searches were limited to articles published in English language.

### 2.2 Study selection

Articles were considered eligible if they met the following inclusion criteria: cohort or case–control studies; female patients diagnosed with breast cancer; patients had diabetes before breast cancer diagnosis; and studies reported at least age-adjusted hazard ratios (HRs) and 95% confidence intervals (CIs) for OS, DFS, or RFP between patients with and without diabetes. The primary end point was the OS and the secondary end points were DFS and RFP. OS was defined as the time from diagnosis or surgery to death due to any cause or last follow-up visit. DFS was defined as the time from diagnosis or surgery to any breast cancer recurrence or death. RFP was defined as the time from diagnosis or surgery to any recurrence. Diabetes mellitus was ascertained by the medical records, antidiabetic medication history, and/or laboratory tests (fasting plasma glucose ≥7.0 mmol/L or a random plasma glucose ≥11.1 mmol/L, or a 2-hour plasma glucose ≥11.1 mmol/L during an oral glucose tolerance test). We excluded case–control or cross-sectional studies or articles that reported unadjusted HR with its 95% CI. Data from reviews, conference abstracts, comments, or duplicated publication also were excluded.

### 2.3 Data extraction and quality assessment

To ensure the accuracy of the extracted data, 2 reviewers (X-BZ and G-SR) independently extracted the data from all included articles. The extracted data included the following: surname of the first author, publication year, study design, study origin, number of breast cancer patients, age of patients, method of diabetes ascertainment, number of patients with diabetes, multivariable adjusted HR and 95% CI, duration of follow-up, and adjusted covariates in the statistical analysis. The Newcastle–Ottawa scale (NOS) for the cohort studies was used to assess the methodological quality of the included studies.^[13]^ Overall NOS scores range from 0 to 9 and studies with scores of 7 to 9 are considered as high quality. Any discrepancies in data extraction and quality assessment were resolved by consensus.

### 2.4 Statistical analysis

All meta-analyses were conducted using Stata version 12.0 statistical software (Stata, College Station, TX). The impact of diabetes on the prognosis of breast cancer patients was expressed as an HR and its 95% CI. The low CI and upper CI for the HR were logarithmically transformed to stabilize the variance and normalize their distribution. The pooled HR was used to examine the association of diabetes with OS, DFS, and RFP in breast cancer patients. A summary HR >1 indicated a worse prognosis in patients with diabetes. Heterogeneity across studies was evaluated using the I^2^ statistic and Cochran Q test. The I^2^ statistic ≥50% or Cochran Q test *P* value <0.10 indicated a significant heterogeneity.^[14]^ When there was significant heterogeneity across studies, a random-effects model was selected in the pooled analysis; otherwise, a fixed-effects model was applied. Subgroup analyses were performed by the origin of study (Asia vs Europe + North America), study design (prospective cohort vs retrospective cohort vs case–control), definition of diabetes (with or without laboratory test), follow-up duration (>5 vs ≤5 years), number of breast cancer patients (≥1000 vs <1000), and NOS scores (≥6 vs <6 stars). Publication bias was assessed by the Begg's rank correlation test^[15]^ and Egger's regression test.^[16]^ Sensitivity analyses were carried out by removing a single study in each turn to investigate the impact of individual studies on the pooled summary estimates.

## 3 Results

### 3.1 Search results and study characteristics

We identified 1372 relevant records through the initial medical electronic searches.

**Figure 1:**
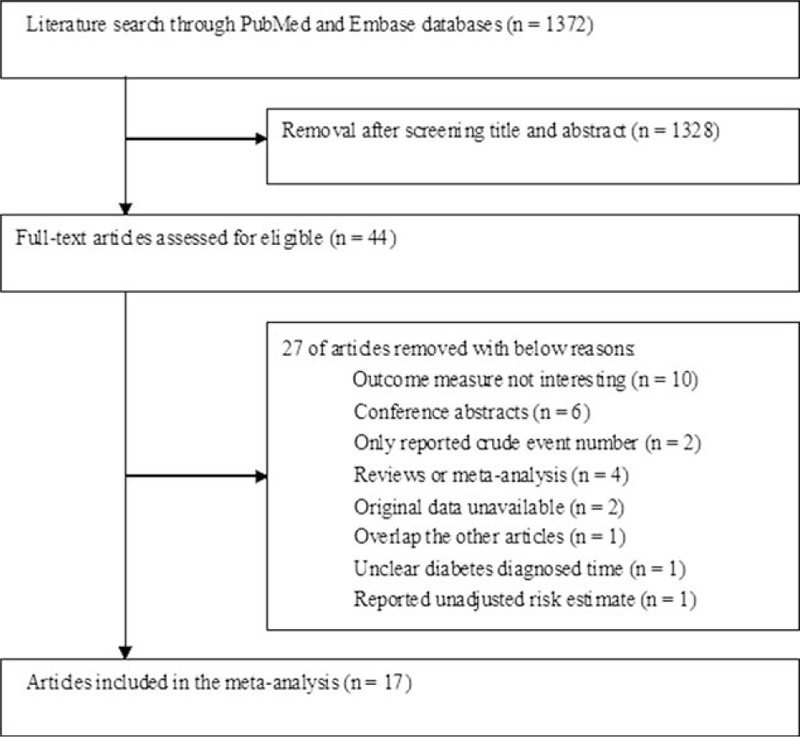
Flowchart of study selection process.

Based on the screening of titles and abstracts, 1328 articles were excluded because they contained overlapping records; obviously irrelevant studies, reviews, or meta-analyses; or an outcome not interesting. Of the remaining 44 records, 27 were further removed after reviewing the full-text articles. Finally, 17 articles^[17–33]^ met our predefined inclusion criteria. Figure 1 shows a flow diagram of the selection process. The characteristics of the 17 included articles are summarized in Table 1. All of the included articles were published from 2001 to 2016. Of the 17 articles, 3 studies^[26,28,32]^ had a prospective cohort design, 10 studies^[17–23,27,29,30]^ had a retrospective cohort design, and 4 studies^[24,25,31,33]^ had a case–control design. The total number of breast cancer patients was 48,315, with study populations ranging from 378 to 6342 in the individual articles. The duration of follow-up ranged from 32 months to 10.3 years. Diabetes was ascertained by medical records, use of antidiabetic medications, self-report, and/or monitoring glucose status. The mean NOS score of the 17 included articles was 6.26 (range, 5–8) stars.

**Table 1:**
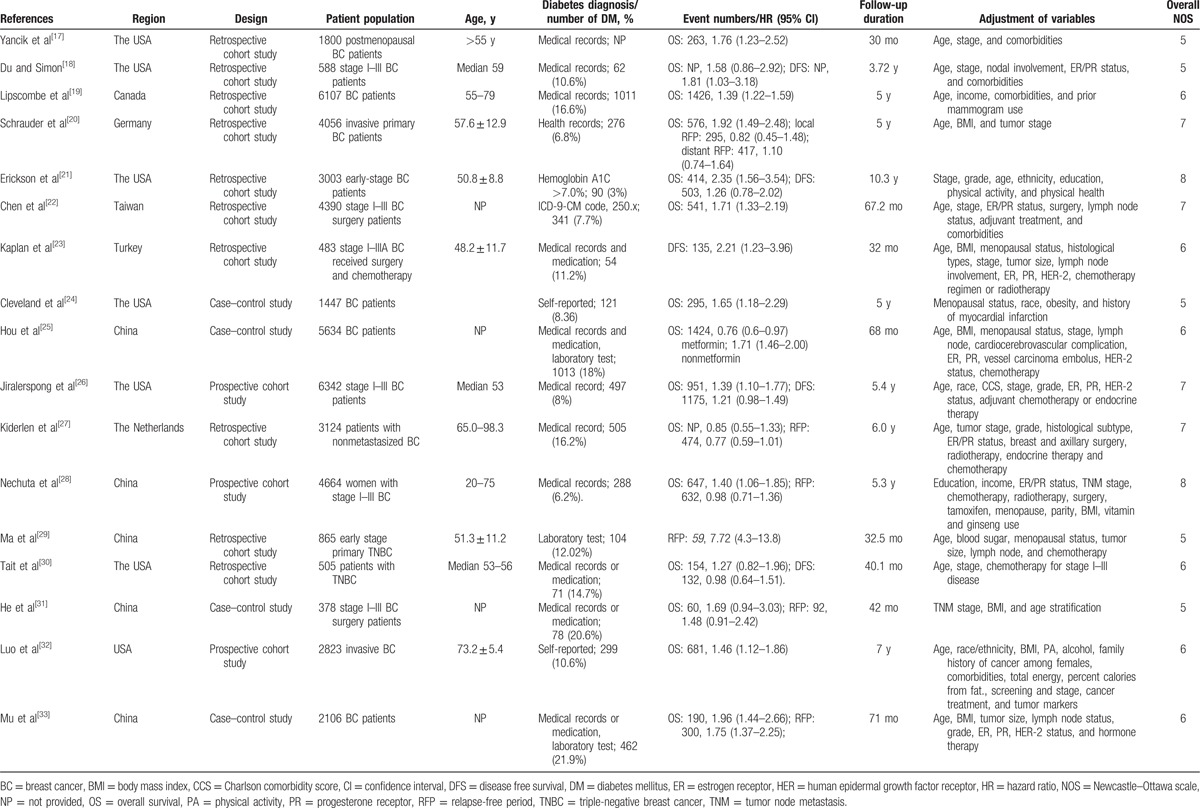
Summary of characteristics of the included studies.

### 3.2 Overall survival

**Figure 2:**
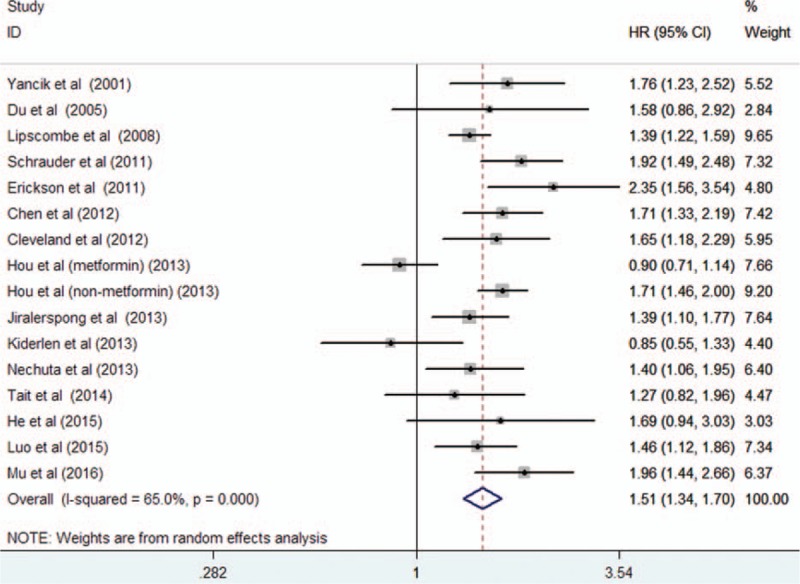
Forest plots showing HR and 95% CI of overall survival in female breast cancer patients with diabetes compared with those without diabetes. CI = confidence interval, HR = hazard ratio.

**Figure 3:**
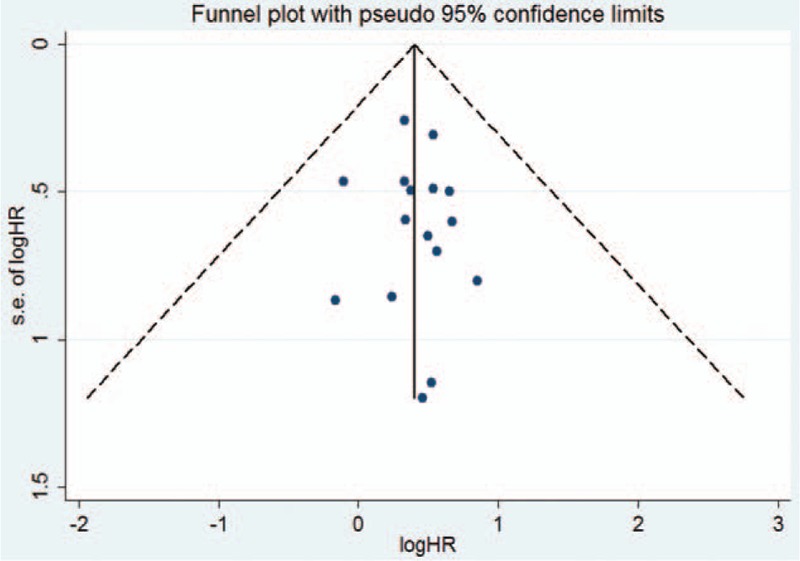
Funnel plots based on overall survival. HR = hazard ratio, SE = standard error.

Fifteen studies^[17–22,24–28,30–33]^ reported the effect of diabetes on the OS in female breast cancer patients. The study by Hou et al^[25]^ provided risk estimate of OS with metformin or without metformin treatment; we therefore pooled the risk estimate, separately. As shown in Fig. 2, a random-effects model was applied because heterogeneity across the studies was significant (I^2^ = 65.0%; *P* < 0.001). The pooled analysis showed that breast cancer patients with diabetes had a shorter OS (HR 1.51; 95% CI 1.34–1.70) than those without diabetes. We did not find evidences of publication bias according to the funnel plots (Fig. 3), Begg's test (*P* = 0.822), and Egger's test (*P* = 0.799). Sensitivity analyses by sequential removal of individual studies did not change the significance of the overall summary estimate. Subgroup analyses indicated that the origin of the study, definition of diabetes, follow-up duration, number of sample sizes, and study design did not obviously affect the prognostic effect of diabetes (Table 2).

**Table 2:**
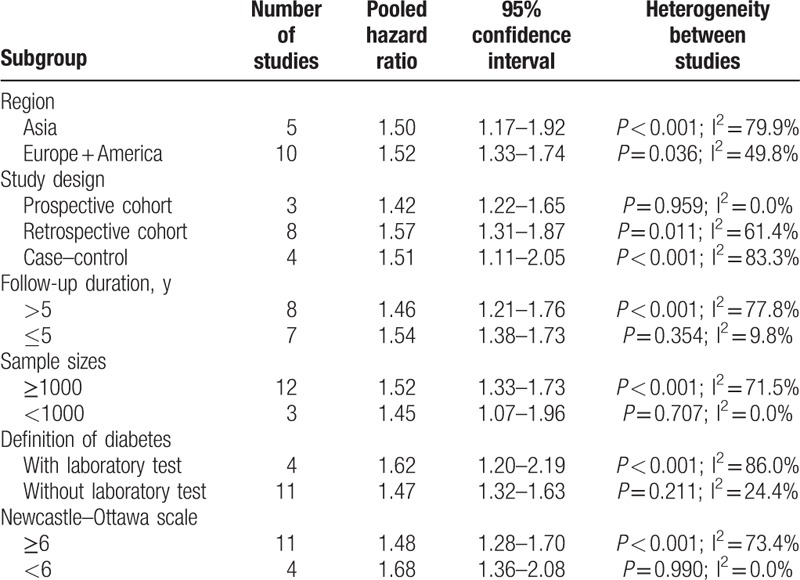
Subgroup analyses on overall survival.

### 3.3 Disease-free survival

**Figure 4:**
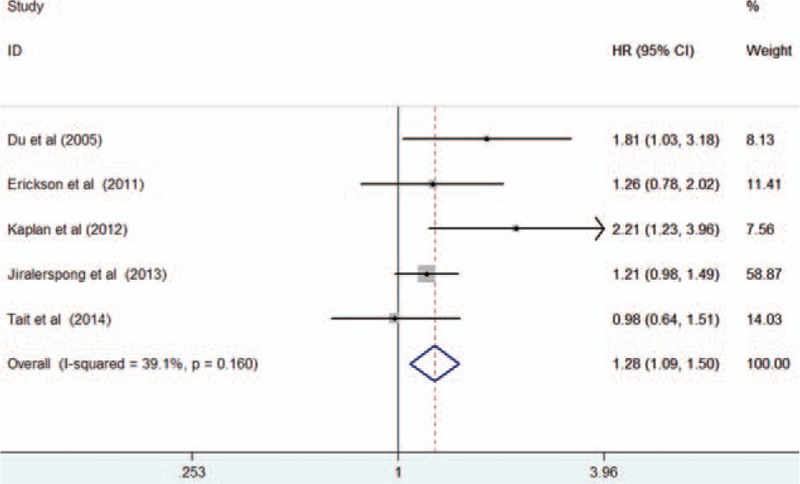
Forest plots showing HR and 95% CI of disease-free survival in female breast cancer patients with diabetes compared with those without diabetes. CI = confidence interval, HR = hazard ratio.

Five studies^[18,21,23,26,30]^ reported an association between diabetes and DFS. As shown in Fig. 4, the pooled HR for DFS was 1.28 (95% CI 1.09–1.50; I^2^ = 39.1%; *P* = 0.160) comparing breast cancer patients with diabetes to those without diabetes in a fixed-effects model.

### 3.4 Relapse-free period

**Figure 5:**
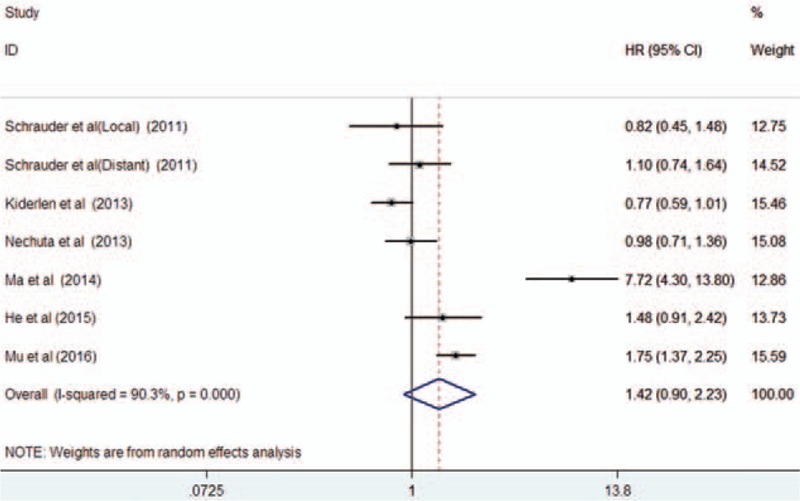
Forest plots showing HR and 95% CI of relapse-free period in female breast cancer patients with diabetes compared with those without diabetes. CI = confidence interval, HR = hazard ratio.

**Figure 6:**
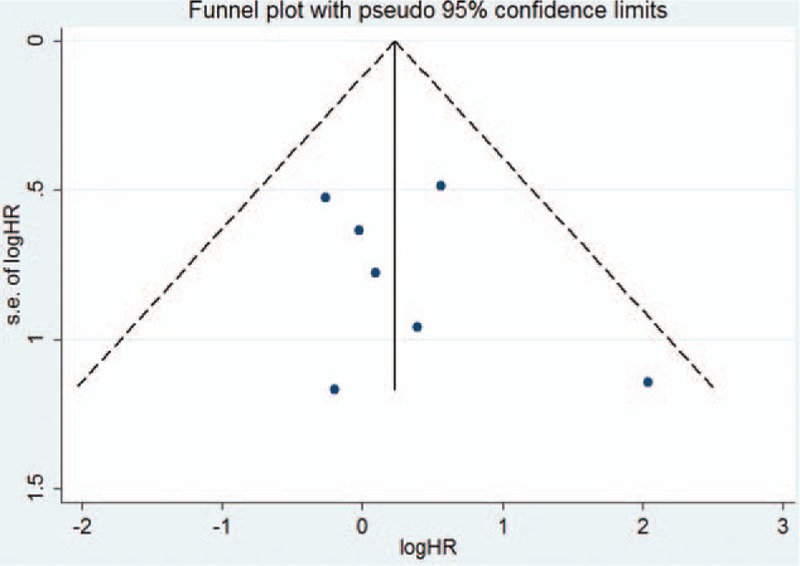
Funnel plots based on relapse-free period. HR = hazard ratio, SE = standard error.

Six studies^[20,27–29,31,33]^ reported an association between diabetes and RFP. As shown in Fig. 5, a random-effects model was selected because heterogeneity across the studies was significant (I^2^ = 90.3%; *P* < 0.001). The pooled HR for RFP was 1.42 (95% CI 0.90–2.23) comparing breast cancer patients with diabetes to those without diabetes. Funnel plots (Fig. 6), Begg's test (*P* = 0.548), and Egger's test (*P* = 0.481) did not reveal the evidence of publication bias. Sensitivity analyses showed that removal of any single study did not have a significant impact on the overall summary estimates (data not shown).

## 4 Discussion

The present meta-analysis is the first to assess the association between diabetes mellitus and the prognosis of women with breast cancer in terms of DFS and RFP. Our meta-analysis showed that breast cancer patients with preexisting diabetes had a 51% shorter OS time and 28% shorter DFS compared with their nondiabetic counterparts. However, the effect of preexisting diabetes on RFP appeared to be not obvious in female breast cancer patients. The current meta-analysis reinforces the effect of diabetes as an independent risk factor for survival outcome in female breast cancer patients.

There have been no consensuses on international consensus standards for the definition of survival end points. In the current study, OS was defined as the time from diagnosis or surgery to death due to any cause or last follow-up visit. DFS was defined as the time from diagnosis or surgery to any recurrence (local or regional) and death due to any cause. RFP was defined as the time from diagnosis or surgery to any disease recurrence, but death was not included. DFS and RFP events occurred more quickly and frequently than OS events. RFP may be identified as a reliable breast cancer–specific end point, particularly in elderly breast cancer patients. We found that diabetes was associated with a shorter OS time for breast cancer patients, but the impact of diabetes on RFP was not statistically significant. This lack of statistical significance in RFP however could be a matter of power due to the fact that fewer studies were included in their analyses. The increased mortality risk is closely correlated to disease recurrences or relapse. In addition to diabetes and its complications, other comorbidities also had a larger impact on survival in breast cancer patients,^[34]^ and may affect the true impact of diabetes on OS.

There was substantial heterogeneity (I^2^ = 65.0%; *P* < 0.001) in the pooling risk estimate of the primary end point OS. Subgroup analyses based on follow-up duration showed that significant heterogeneity was found in studies with follow-up duration >5 years (I^2^ = 77.8%) but not in the studies with a <5-year follow-up (I^2^ = 9.8%). In the sensitivity analysis, when we removed 2 studies,^[25,27]^ there was no obvious heterogeneity (I^2^ = 20.4%; *P* = 0.238). Therefore, the type of hypoglycemic agents used and the older age of patients (>65 years) may be major sources of significant heterogeneity.

Both diabetes and breast cancer are the major causes of morbidity and mortality worldwide.^[35]^ The prognosis was found to be poor in patients with coexistent diabetes and breast cancer patients. An important issue is the impact of antihyperglycemic agents on the prognosis of breast cancer patients. Intensive or loose glycemic control may affect the prognosis of breast cancer patients. Metformin therapy has been demonstrated to decrease all-cause mortality in patients with breast cancer.^[11]^ The impact of glycemic control and/or modification with antihyperglycemic agents on the prognosis of breast cancer patients needs to be further explored.

The potential interplay between diabetes and breast cancer prognosis is complex. Diabetes can directly influence breast cancer prognosis by altering hyperinsulinemia and insulin-like growth factors, endogenous sex hormones, and inflammatory markers.^[21]^ Hyperinsulinemia and insulin-like growth factors may play roles in promoting breast cancer development.^[36,37]^ Chronic proinflammatory conditions and oxidative stress induced by impaired glucose metabolism were considered to promote tumor initiation and progression.^[38]^ Clinically, diabetes in breast cancer patients could increase the risk of chemotherapy-related toxicity,^[5]^ resulting in the administration of receiving less aggressive treatments or poorer responses to cancer treatment.^[39]^ Less intensive care for diabetes and/or cancer care could negatively affect the prognosis of breast cancer patients with diabetes.

Limitations of the present meta-analysis should be mentioned. First, our findings were based on study-level data and not on individual patient data. Individual patient data could provide more reliable summary estimates of the association. Furthermore, most of the included studies (14/17 articles) were retrospective in nature and potential selection bias or recall bias cannot be excluded. More well-designed prospective studies are necessary in future investigations. Second, substantial heterogeneity was observed in the pooled OS and RFP. The observed significant heterogeneity may be partly attributed to patients’ characteristics, the method used for diabetes ascertainment, and length of follow-up, or because the study design may partly contribute to the observed heterogeneity. Third, the included studies did not adjust for confounding factors in a consistent way, and residual confounding factors could lead to overestimation of the risk estimates. Finally, the use of antidiabetic medication and intensive glycemic control was not taken into account to analyze the impact of diabetes on breast cancer prognosis.

## 5 Conclusions

The present meta-analysis reveals that preexisting diabetes is an independent predictor of poor OS and DFS, even after adjustment for multiple confounding variables, in female breast cancer patients. However, the impact of diabetes on RFP needs to be further verified. Future well-designed prospective studies should investigate whether intensive glycemic control and/or modification with antihyperglycemic agents can lead to improvement in the prognosis of breast cancer patients with diabetes.
